# The wild bootstrap for multivariate Nelson–Aalen estimators

**DOI:** 10.1007/s10985-018-9423-x

**Published:** 2018-03-06

**Authors:** Tobias Bluhmki, Dennis Dobler, Jan Beyersmann, Markus Pauly

**Affiliations:** 1 Institute of Statistics, Ulm University, Helmholtzstrasse 20, 89081 Ulm, Germany; 2 Department of Mathematics, Vrije Universiteit Amsterdam, De Boelelaan 1081a, 1081 HV Amsterdam, The Netherlands

**Keywords:** Conditional central limit theorem, Counting process, Equivalence test, Proportional hazards, Kolmogorov–Smirnov test, Survival analysis, Weak convergence

## Abstract

We rigorously extend the widely used wild bootstrap resampling technique to the multivariate Nelson–Aalen estimator under Aalen’s multiplicative intensity model. Aalen’s model covers general Markovian multistate models including competing risks subject to independent left-truncation and right-censoring. This leads to various statistical applications such as asymptotically valid confidence bands or tests for equivalence and proportional hazards. This is exemplified in a data analysis examining the impact of ventilation on the duration of intensive care unit stay. The finite sample properties of the new procedures are investigated in a simulation study.

**Electronic supplementary material:**

The online version of this article (10.1007/s10985-018-9423-x) contains supplementary material, which is available to authorized users.

## Introduction

One of the most crucial quantities within the analysis of time-to-event data with independently right-censored and left-truncated survival times is the cumulative hazard function, also known as cumulative transition intensity. Most commonly, it is nonparametrically estimated by the well-known *Nelson–Aalen estimator* (Andersen et al. 1993, Chapter IV). In this context, time-simultaneous confidence bands are the perhaps best interpretative tool to account for related estimation uncertainties.

The construction of confidence bands is typically based on the asymptotic behavior of the underlying stochastic processes, more precisely, the (properly standardized) Nelson–Aalen estimator asymptotically behaves like a Wiener process. Early approaches utilized this property to derive confidence bands for the cumulative hazard function; see e.g., Bie et al. (1987) or Section IV.1.3 in Andersen et al. (1993).

However, Dudek et al. (2008) found that this approach applied to small samples can result in considerable deviations from the nominal level. To improve small sample properties, Efron (1979, 1981) suggested a computationally convenient and flexible resampling technique, called *bootstrap*, where the unknown non-Gaussian quantile is approximated via repeated generation of point estimates based on random samples of the original data. For a detailed discussion within the standard right-censored survival setup, see also Akritas (1986), Lo and Singh (1986), and Horvath and Yandell (1987). The simulation study of Dudek et al. (2008) particularly reports improvements of bootstrap-based confidence bands for the hazard function as compared to those using asymptotic quantiles. An alternative is the so-called *wild bootstrap* firstly proposed in the context of regression analyses (Wu 1986). As done in Lin et al. (1993), the basic idea is to replace the (standardized) residuals with independent standardized variates—so-called multipliers—while keeping the data fixed. One advantage compared to Efron’s bootstrap is to gain robustness against variance heteroscedasticity (Wu 1986). Using standard normal multipliers, this resampling procedure has been applied to construct time-simultaneous confidence bands for survival curves under the Cox proportional hazards model (Lin et al. 1994) and adapted to cumulative incidence functions in the competing risks setting (Lin 1997). The latter approach has recently been extended to general wild bootstrap multipliers with mean zero and variance one (Beyersmann et al. 2013), which indicate possible improved small sample performances. This result was confirmed in Dobler and Pauly (2014) as well as Dobler et al. (2017), where more general resampling schemes are discussed. In all these references, only one multiplier, typically standard normal, was required per individual, because each individual experienced at most one event. If an individual may experience more than one event, multiple multipliers per individual were, e.g., considered by Dabrowska and Ho (2000) in the context of Cox modelling, see also Shu et al. (2007) for Cox models in a semi-Markov illness-death model, and Liu et al. (2008) in a progressive multistate model for current leukemia free survival.

The present article focuses on the nonparametric estimation of cumulative hazard functions and proposes a general and flexible wild bootstrap resampling technique, which is valid for a large class of time-to-event models. In particular, the procedure is not limited to the standard survival or competing risks framework. The key assumption is that the involved counting processes satisfy the so-called *multiplicative intensity model* (Andersen et al. 1993). Consequently, arbitrary Markovian multistate models with finite state space are covered, as well as various other intensity models (e.g., excess or relative mortality models, cf. Andersen and Væth 1989) and specific semi-Markov situations (Andersen et al. 1993, Example X.1.7). Independent right-censoring and left-truncation can straightforwardly be incorporated.

The main aim of this article is to mathematically justify the wild bootstrap technique for the multivariate Nelson–Aalen estimator in this general framework, while using not necessarily normally distributed but possibly multiple multipliers per counting process in the resampling step, This is accomplished by a novel martingale-based proof that discloses the close connection between the estimator and its wild bootstrap version. This insight would not have been possible by generalizing the elementary approach for showing bootstrap validity in competing risks given in Beyersmann et al. (2013) and Dobler and Pauly (2014) to the present set-up. Compared to the standard survival or competing risks setting, with at most one transition per individual, the major difficulty is to account for counting processes having an arbitrarily large random number of jumps. We will see that utilizing only one multiplier per individual counting process leads to the wrong covariance structure in general; instead, one multiplier per increment is required. As Beyersmann et al. (2013) suggested in the competing risks setting, we also allow for more general multipliers with expectation 0 and variance 1 and extend the resulting weak convergence theorems to resample the multivariate Nelson–Aalen estimator in our general setting. For practical applications, this result allows, for instance, within- or two-sample comparisons and the formulation of statistical tests.

The wild bootstrap is exemplified to statistically assess the impact of mechanical ventilation in the intensive care unit (ICU) on the length of stay. A related problem is to investigate ventilation-free days, which was established as an efficacy measure in patients subject to acute respiratory failure (Schoenfeld et al. 2002). However, applications of their methodology (see e.g., Sauaia et al. 2009; Stewart et al. 2009) rely on the constant hazards assumption. Other publications like de Wit et al. (2008), Trof et al. (2012), or Curley et al. (2015) used a Kaplan–Meier-type procedure that does not account for the more complex multistate structure. In contrast, we propose an illness-death model with recovery that methodologically works under the more general time-inhomogeneous Markov assumption and captures both the time-dependent structure of mechanical ventilation and the competing endpoint ‘death in ICU’.

The remainder of this article is organized as follows: Sect. 2 introduces cumulative hazard functions and their Nelson–Aalen estimators using counting process formulations. After summarizing its asymptotic properties, Sect. 3 offers our main theorem on conditional weak convergence for the wild bootstrap. This allows for various statistical applications in Sect. 4: Two-sided hypothesis tests and various sorts of time-simultaneous confidence bands are deduced, as well as simultaneous confidence intervals for a finite set of time points. Furthermore, tests for equivalence, inferiority and superiority as well as for proportionality of two hazard functions constitute useful criteria in practical data analyses. A simulation study assessing small and large sample performances of both the derived confidence bands in comparison to the algebraic approach based on the time-transformed Brownian motion and the tests for proportional hazards is reported in Sect. 5. The SIR-3 data on patients in ICU (Beyersmann et al. 2006; Wolkewitz et al. 2008) serves as its template and is practically revisited in Sect. 6. Concluding remarks and a discussion are given in Sect. 7. All proofs are deferred to Appendix A and the non-applicability of the ordinary multiplier resampling is verified in Appendix B.

## Nonparametric estimation under the multiplicative intensity structure

Throughout, we adopt the notation of Andersen et al. (1993). For ${k\in \mathbb {N}}$, let $\mathbf N =\left( N_1,\ldots ,N_k\right) '$ be a multivariate counting process which is adapted to a filtration $({\mathcal {F}}_t)_{t \ge 0}$. Each entry $N_j, j=1,\dots ,k,$ is supposed to be a càdlàg function, zero at time zero, and to have piecewise constant paths with jumps of size one. In addition, assume that no two components jump at the same time and that each $N_{j}(t)$ satisfies the *multiplicative intensity model* of Aalen (1978) with intensity process given by $\lambda _j(t) = \alpha _j(t) Y_j(t)$. Here, $Y_j(t)$ defines a predictable process not depending on unknown parameters and $\alpha _j$ describes a non-negative (hazard) function. For well-definiteness, the observation of $\mathbf N$ is restricted to the interval $[0,\tau ]$, where $\tau< \tau _j = {\sup }\big \{ u \ge 0 :\int _{(0,u]}\alpha _j(s)ds<\infty \big \} \ \text {for all } j = 1, \dots , k.$ The multiplicative intensity structure covers several customary frameworks in the context of time-to-event analysis. The following overview specifies frequently used models.

### Example 1

- Markovian multistate models with finite state space ${\mathcal {S}}$ are very popular in biostatistics. In this setting, $Y_\ell (t)$ represents the total number of individuals in state $\ell$ just prior to *t* (‘number at risk’), whereas $\alpha _{\ell m}(t)$ is the instantaneous risk (‘transition intensity’) to switch from state $\ell$ to *m*, where $\ell , m \in {\mathcal {S}}$, $\ell \ne m$. Here, $N_\ell = \sum _{i=1}^n N_{\ell ;i}$ is the aggregation over individual-specific counting processes with $n \in \mathbb {N}$ individuals under study. For specific examples (such as competing risks or the illness-death model) and details including the incorporation of independent left-truncation and right-censoring, see Andersen et al. (1993) and Aalen et al. (2008).
- Other examples are the relative or excess mortality model, where not all individuals necessarily share the same hazard rate $\alpha$. In this case *Y* cannot be interpreted as the total number of individuals at risk as in part (a); see Example IV.1.11 in Andersen et al. (1993) for details.
- The time-inhomogeneous Markov assumption required in part (a) can even be relaxed in specific situations: Following Example X.1.7 in Andersen et al. (1993), consider an illness-death model without recovery. Assuming that the transition intensity $\alpha _{12}$ depends on the duration *d* in the intermediate state, but not on time *t*, leads to a semi-Markov process not satisfying the multiplicative intensity structure. This is because the intensity process of $N_{12}(t)$ is given by $\alpha _{12}(t-T)Y_1(t)$, where the first factor of the product is not deterministic anymore. Here, *T* is the random transition time into state 1. However, when $d=t-T$ is used as the basic timescale, the counting process $K(d)=N_{12}(d+T)$ has intensity $\alpha _{12}(d)Y_1(d)$ with respect to the filtration
  $$\begin{aligned} {\mathcal {F}}_d=\left( \sigma \lbrace (N_{01}(t), N_{02}(t)): 0<t<\tau \rbrace \vee \sigma \lbrace K(d): 0<d<\infty \rbrace \right) . \end{aligned}$$

Thus, the multiplicative intensity structure is fulfilled.

Under the above assumptions, the Doob–Meyer decomposition applied to $N_j$ leads to

2.1 $$\begin{aligned} dN_{j}(s)= \lambda _{j}(s)ds+dM_{j}(s), \end{aligned}$$

where the $M_{j}$ are zero-mean martingales with respect to $({\mathcal {F}}_t)_{t \in [0,\tau ]}$. The canonical nonparametric estimator of the cumulative hazard function $A_j(t)=\int _{(0,t]}\alpha _j(s)ds$ is given by the so-called *Nelson–Aalen estimator*

$$\begin{aligned} \hat{A}_{jn}(t)=\int \limits _{(0,t]}\frac{J_j(s)}{Y_j(s)}dN_j(s). \end{aligned}$$

Here, $J_j(t)=\mathbf {1}\{Y_j(t)>0\}$, $\frac{0}{0} := 0$, and $n \in \mathbb {N}$ is a sample size-related number (that goes to infinity in asymptotic considerations). Its multivariate counterpart is introduced by $\hat{\mathbf{A}}_{n}:=(\hat{A}_{1n},\ldots ,\hat{A}_{kn})^{\prime }$. As in Andersen et al. (1993), suppose that there exist deterministic functions $y_j$ with $\inf _{u \in [0,\tau ]} y_j(u)>0$ such that

2.2 $$\begin{aligned} \underset{s\in [0,\tau ]}{\sup }\left| \frac{Y_j(s)}{n}-y_j(s)\right| \xrightarrow []{\ P\ }0 \quad \text {for all } j=1,\dots , k , \end{aligned}$$

where ‘${\mathop {\rightarrow }\limits ^{P}}$’ denotes convergence in probability for $n\rightarrow \infty$. For each *j*, define the normalized Nelson–Aalen process $W_{jn}:=\sqrt{n} ( \hat{A}_{jn}-A_j )$ possessing the asymptotic martingale representation

2.3 $$\begin{aligned} W_{jn}(t) \doteqdot \sqrt{n}\int \limits _{(0,t]}\frac{J_j(s)}{Y_j(s)}dM_j(s) \end{aligned}$$

with $M_{j}$ given by (2.1). Here, ‘$\doteqdot$’ means that the difference of both sides converges to zero in probability. Define the vectorial aggregation of all $W_{jn}$ as ${{\varvec{W}}}_n = (W_{1n}, \dots , W_{kn})'$ and let ‘${\mathop {\rightarrow }\limits ^{d}}$’ denote convergence in distribution for $n\rightarrow \infty$. Then, Theorem IV.1.2 in Andersen et al. (1993) in combination with (2.2) provides a weak convergence result on the *k*-dimensional space $\mathfrak {D}[0,\tau ]^k$ of càdlàg functions endowed with the product Skorohod topology.

### Theorem 1

If assumption (2.2) holds, we have convergence in distribution

2.4 $$\begin{aligned} \mathbf W _{n} {\mathop {\longrightarrow }\limits ^{d}} \mathbf U = (U_1, \dots , U_k)', \end{aligned}$$

on $\mathfrak {D}[0,\tau ]^k$, where $U_1, \dots , U_k$ are independent zero-mean Gaussian martingales with covariance functions $\psi _j(s_1,s_2):=Cov(U_j(s_1),U_j(s_2))={\int }_{(0,s_1]}\frac{\alpha _j(s)}{y_j(s)}ds$ for $j = 1, \dots , k$ and $0\le s_1\le s_2\le \tau$.

The covariance function $\psi _j$ is commonly approximated by the *Aalen-type*

2.5 $$\begin{aligned} \hat{\sigma }_j^2(s_1) = n \int \limits _{(0,s_1]} \frac{J_j(s)}{Y_j^2(s)} d N_j(s). \end{aligned}$$

or the *Greenwood-type* estimator

2.6 $$\begin{aligned} \hat{\sigma }_j^2(s_1) = n \int \limits _{(0,s_1]} \frac{J_j(s) (Y_j(s)-\varDelta N_j(s))}{Y_j^3(s)} d N_j(s) \end{aligned}$$

which are consistent for $\psi _j(s_1,s_2)$ under the assumption of Theorem 1; cf. (4.1.6) and (4.1.7) in Andersen et al. (1993). Here, $\varDelta N_j(s)$ denotes the jump size of $N_j$ at time *s*.

## Inference via Brownian bridges and the wild bootstrap

As discussed in Andersen et al. (1993), the limit process ${{\varvec{U}}}$ can analytically be approximated via Brownian bridges. However, improved coverage probabilities in the simulation study in Sect. 5 suggest that the proposed wild bootstrap approach may be preferable. First, we sum up the classic result.

### Inference via transformed Brownian bridges

The asymptotic mutual independence stated in Theorem 1 allows to focus on a single component of ${{\varvec{W}}}_n$, say $W_{1n} = \sqrt{n} ( \hat{A}_{1n} - A_1 )$. For notational convenience, we suppress the subscript 1. Let *g* be a positive (weight) function on an interval $[t_1,t_2]\subset [0,\tau ]$ of interest and $B^0$ a standard Brownian bridge process. Then, as $n \rightarrow \infty$, it is established in Section IV.1 in Andersen et al. (1993) that

3.1 $$\begin{aligned} \sup _{s \in [t_1,t_2]} \Big | \frac{\sqrt{n} ( \hat{A}_n(s) - A(s))}{1 + \hat{\sigma }^2(s)} g \Big (\frac{\hat{\sigma }^2(s)}{1 + \hat{\sigma }^2(s)} \Big ) \Big | {\mathop {\longrightarrow }\limits ^{d}} \sup _{s \in [\phi (t_1),\phi (t_2)]} | g(s) B^0(s) |. \quad \end{aligned}$$

Here $\phi (t) = \frac{\sigma ^2(t)}{1 + \sigma ^2(t)}$, $\sigma ^2(t) = \psi (t,t)$ and $\hat{\sigma }^2(t)$ is a consistent estimator for $\sigma ^2(t)$, such as (2.5) or (2.6). Quantiles of the right-hand side of (3.1) for $g\equiv 1$ are recorded in tables (e.g., Koziol and Byar 1975; Hall and Wellner 1980; Schumacher 1984). For general *g*, they can be approximated via standard statistical software.

Even though relation (3.1) enables statistical inference based on the asymptotics of a central limit theorem, appropriate resampling procedures usually showed improved properties; see e.g., Hall and Wilson (1991), Good (2005) and Pauly et al. (2015).

### Wild bootstrap resampling

In contrast to, for instance, a competing risks model where each counting process $N_{j}$ is at most *n*, the number $N_{j}(\tau )$ is not necessarily bounded in our setup only assuming Aalen’s multiplicative intensity model. Hence, a modification of the multiplier resampling scheme under competing risks suggested by Lin (1997) and elaborated by Beyersmann et al. (2013) is required. For this purpose, introduce counting process-specific stochastic processes indexed by $s \in [0,\tau ]$ that are independent of $N_j, Y_j$ for all $j=1,\dots ,k$. Let $(G_j(s))_{s \in [0,\tau ]}, 1 \le j \le k,$ be independently and identically distributed (i.i.d.) white noise processes such that each $G_j(s)$ satisfies $\mathbb {E}(G_j(s)) = 0$ and $var(G_j(s))=1$, $j=1,\dots ,k$, $s \in [0,\tau ]$. That is, all $\ell$-dimensional marginals of $G_1$, $\ell \in \mathbb {N}$, shall be the same $\ell$-fold product-measure. Then, a *wild bootstrap version* of the normalized multivariate Nelson–Aalen estimator ${{\varvec{W}}}_n$ is defined as

3.2 $$\begin{aligned} \hat{{{\varvec{W}}}}_{n}(t)= & {} (\hat{W}_{1n}(t), \dots , \hat{W}_{kn}(t) )' \nonumber \\:= & {} \sqrt{n} \bigg ( \underset{(0,t]}{\int }\frac{J_1(s)}{Y_1(s)}G_{1}(s) dN_{1}(s), \dots , \underset{(0,t]}{\int }\frac{J_k(s)}{Y_k(s)} G_{k}(s) dN_{k}(s) \bigg )'. \end{aligned}$$

In words, $\hat{{{\varvec{W}}}}_{n}$ is obtained from representation (2.3) of ${{\varvec{W}}}_n$ by substituting the unknown individual martingale processes $M_{j}$ with the *observable* quantities $G_{j} N_{j}$. Even though only the values of each $G_j$ at the jump times of $N_j$ are relevant, this construction in terms of white noise processes enables a consideration of the wild bootstrap process on a product probability space; see the Appendix for details.

Consider for a moment the special case of a multistate model with *n* i.i.d. individuals (Example 1(a)). For instance, the competing risks model in Lin (1997) involves at most one transition (and thus one multiplier) per individual, while Glidden (2002) introduces only one multiplier per individual for estimating state occupation probabilities in general non-Markov multistate models. In contrast, our resampling approach is a new approach in the sense that it involves independent weightings of all jumps even within the same individual. The consequence is that, instead of considering one multiplier per individual, we need to utilize a white noise processes as done in (3.2) in order to account for randomly many numbers of events per individual.

The limit distribution of $\hat{{{\varvec{W}}}}_n$ may be approximated by simulating a large number of replicates of the *G*’s, while the data is kept fixed. For a competing risks setting with standard normally distributed multipliers, our general scheme reduces to the one discussed in Lin (1997).

For the remainder of the paper, we summarize the available data in the $$\sigma $$-algebra $$\mathcal {C}_0 = \sigma \{N_{j}(u),$$$$Y_{j}(u):j = 1,\dots ,k,\ u\in [0,\tau ]\}.$$ A natural way to introduce a filtration based on $${\mathcal {C}}_0$$ that progressively collects information on the white noise processes is by setting

$$\begin{aligned} \mathcal {C}_t = {\mathcal {C}}_0 \ \vee \ \sigma \{ G_j(s): j=1,\dots ,k, \ s \in [0,t] \}. \end{aligned}$$

The following lemma is a key argument in an innovative, martingale-based consistency proof of the proposed wild bootstrap technique.

#### Lemma 1

For each $n \in \mathbb {N}$, the wild bootstrap version of the multivariate Nelson–Aalen estimator $(\hat{{{\varvec{W}}}}_{n}(t))_{t \in [0,\tau ]}$ is a square-integrable martingale with respect to the filtration $(\mathcal {C}_t)_{t \in [0,\tau ]}$ with orthogonal components. Its predictable variation process is given by

$$\begin{aligned} \langle \hat{{{\varvec{W}}}}_{n} \rangle : \ t \ \longmapsto \ n \bigg ( \int _0^t \frac{J_1(s)}{Y_1^2(s)} d N_1(s), \dots , \int _0^t \frac{J_k(s)}{Y_k^2(s)} d N_k(s) \bigg ) \end{aligned}$$

and its optional variation process by

$$\begin{aligned} {[}\hat{{{\varvec{W}}}}_{n} ] : \ t \ \longmapsto \ n \bigg ( \int _0^t \frac{J_1(s)}{Y_1^2(s)} G_1^2(s) d N_1(s), \dots , \int _0^t \frac{J_k(s)}{Y_k^2(s)} G_k^2(s) d N_k(s) \bigg ) . \end{aligned}$$

The following conditional weak convergence result justifies the approximation of the limit distribution of ${{\varvec{W}}}_{n}$ via $\hat{{{\varvec{W}}}}_n$ given $\mathcal {C}_0$. Both, the general framework requiring only Aalen’s multiplicative intensity structure as well as using possibly non-normal multipliers are original to the present paper.

#### Theorem 2

Let $\mathbf U$ be as in Theorem 1. Assuming (2.2), we have the following conditional convergence in distribution on $\mathfrak {D}[0,\tau ]^k$ given $\mathcal {C}_0$ as $n \rightarrow \infty$:

$$\begin{aligned} \hat{\mathbf{W }}_{n}{\mathop {\longrightarrow }\limits ^{d}} \mathbf U \quad \text {in probability.} \end{aligned}$$

#### Remark 1

- Reconsider the ordinary multiplier resampling based on a sequence of (time-constant) i.i.d. random variables $D_1, \dots , D_n$ with $E(D_1) = 0$ and unit variance where, in the resampling step, the martingales $M_j$ are replaced with $D_j N_j$. In contrast to the wild bootstrap based on white noise processes, the wild bootstrap using the time-constant sequence $D_1, \dots , D_n$ fails to reproduce the correct covariance structure of the Nelson–Aalen process. Even in the special univariate Markovian case, the limit process does not have independent increments and it hence necessarily differs from the asymptotics described in Theorem 2; see Appendix B for details.
- It is due to the martingale property of the wild bootstrapped multivariate Nelson–Aalen estimator that we anticipate a good finite sample approximation of the unknown distribution of the Nelson–Aalen estimator. In particular, the wild bootstrap, realized by white noise processes as above, succeeds in imitating the martingale structure of the original Nelson–Aalen estimator. The predictable variation process of the wild bootstrap process equals the optional variation process of the centered Nelson–Aalen process. Hence, both processes share the same properties and approximately the same covariance structure.
- Suppose that $E(n^k J_1(u) / Y_1^k(u)) = O(1)$ for some $k \in \mathbb {N}$ and all $u \in [0,\tau ]$, which for example holds for any $k \in \mathbb {N}$ if $Y_1$ has a number at risk interpretation. Since different increments of ${{\varvec{W}}}_n$ (to arbitrary powers) are uncorrelated, it can be shown that the convergence in Theorem 1 for single $t \in [0,\tau ]$ even holds in the Mallows metric $d_p$ for any $0 < p \le k$; see, for instance, Bickel and Freedman (1981) for such theorems related to the classical bootstrap. Provided that the *r*th moment of $G_1(u)$ exists, similar arguments show that the convergence in probability in Theorem 2 for single $t \in [0,\tau ]$ holds in the Mallows metric $d_p$ for any even $0 < p \le r$ as well. This of course includes white noise processes with centered *Poi*(1) or standard normal marginals, as applied later on.

## Statistical applications

Throughout this section denote by $\alpha \in (0,1)$ the nominal level of all inference procedures.

### Confidence bands

After having established all required weak convergence results, we discuss different possibilities for realizing confidence bands for $A_j$ around the Nelson–Aalen estimator $\hat{A}_{jn}$, $j=1,\ldots ,k,$ on an interval $[t_1, t_2] \subset [0,\tau ]$ of interest. Later on, we propose a confidence band for differences of cumulative hazard functions. As in Sect. 3.1, we first focus on $A_1$ and suppress the index 1 for notational convenience. Following Andersen et al. (1993), Section IV.1, we consider weight functions

$$\begin{aligned} g_1(s) = (s(1-s))^{-1/2} \qquad \text {or} \qquad g_2 \equiv 1 \end{aligned}$$

as choices for *g* in relation (3.1). The resulting confidence bands are commonly known as *equal precision* and *Hall–Wellner* bands, respectively. We apply a log-transformation in order to improve small sample level $\alpha$ control. Combining the previous sections’ convergences with the functional delta-method and Slutsky’s lemma yields

#### Theorem 3

Under condition (2.2), for any $0 \le t_1 \le t_2 \le \tau$ such that $A(t_1) > 0$, we have the following convergences in distribution on the càdlàg space $\mathfrak {D}[t_1,t_2]$:

4.1 $$\begin{aligned}&\Big ( \sqrt{n} \hat{A}_n \frac{\log \hat{A}_n - \log A}{1 + \hat{\sigma }^2} \Big ) \cdot g \circ \frac{\hat{\sigma }^2}{1 + \hat{\sigma }^2} {\mathop {\longrightarrow }\limits ^{d}} (g B^0) \circ \phi \quad \text {and} \end{aligned}$$

4.2 $$\begin{aligned}&\Big ( \frac{\hat{W}_n}{1 + \sigma ^{*2}} \Big ) \cdot g \circ \frac{\sigma ^{*2}}{1 + \sigma ^{*2}} {\mathop {\longrightarrow }\limits ^{d}} (g B^0) \circ \phi \end{aligned}$$

conditionally given $\mathcal {C}_0$ in probability, with $\phi$ as in Sect. 3 and the wild bootstrap variance estimator $\sigma ^{*2}(s):=n\int _{(0,t]}J(s)Y^{-2}(s)G^2(s)$*dN*(*s*).

In particular, $\sigma ^{*2}$ is a uniformly consistent estimate for $\sigma ^2$ (Dobler and Pauly 2014) and, being the optional variation process of the wild bootstrap Nelson–Aalen process, it may be one natural choice for variance estimation. For practical purposes, we adapt the approach of Beyersmann et al. (2013) and estimate $\sigma ^2$ based on the empirical variance of the wild bootstrap quantities $\hat{{W}}_{n}$. The continuity of the supremum functional translates (4.1) and (4.2) into weak convergences for the corresponding suprema. Hence, the consistency of the following critical values is ensured:

$$\begin{aligned} c_{1-\alpha }^g= & {} (1-\alpha ) \text { quantile of} \quad \mathfrak {L} \left( \sup _{s \in [t_1,t_2]} | g(\hat{\phi }(s)) B^0(\hat{\phi }(s)) | \right) , \\ \tilde{c}_{1-\alpha }^g= & {} (1-\alpha ) \text { quantile of} \quad \mathfrak {L} \left( \sup _{s \in [t_1,t_2]} \Big | \frac{\hat{W}_n(s)}{1 + \sigma ^{*2}(s)} g \left( \frac{\sigma ^{*2}(s)}{1 + \sigma ^{*2}(s)} \right) \Big | \Big | \ \mathcal {C}_0 \right) , \end{aligned}$$

where $\mathfrak {L}(\cdot )$ denotes the law of a random variable. Here, *g* equals either $g_1$ or $g_2$ and $\hat{\phi } = \frac{\hat{\sigma }^2}{1+\hat{\sigma }^2}$. Note, that $\tilde{c}_{1-\alpha }^g$ is, in fact, a random variable. The results are back-transformed into four confidence bands for *A* abbreviated with *HW* and *EP* for the Hall–Wellner and equal precision bands and *a* and *w* for bands based on quantiles of the asymptotic distribution and the wild bootstrap, respectively. In our simulation studies these bands are also compared with the linear confidence band $CB_{dir}^w$, which is based on the critical value

$$\begin{aligned} \quad \tilde{c}_{1-\alpha }= & {} (1-\alpha ) \text { quantile of} \quad \mathfrak {L} \Big ( \sup _{s \in [t_1,t_2]} \big | \hat{W}_n(s) \big | \Big | \ \mathcal {C}_0 \Big ). \end{aligned}$$

#### Corollary 1

Under the assumptions of Theorem 3, the following bands for the cumulative hazard function $(A(s))_{s \in [t_1,t_2]}$ provide an asymptotic coverage probability of $1-\alpha$:

4.3 $$\begin{aligned} \textit{CB}_{\textit{EP}}^a= & {} \left[ \hat{A}_n(s) \exp \left( \mp \frac{c_{1-\alpha }^{g_1}}{\sqrt{n} \hat{A}_n(s)} \hat{\sigma }_n(s) \right) \right] _{s \in [t_1,t_2]} \nonumber \\ \textit{CB}_{\textit{HW}}^a= & {} \left[ \hat{A}_n(s) \exp \left( \mp \frac{c_{1-\alpha }^{g_2}}{\sqrt{n} \hat{A}_n(s)} \Big (1+\hat{\sigma }_n^2(s)\Big ) \right) \right] _{s \in [t_1,t_2]} \nonumber \\ \textit{CB}_{\textit{EP}}^w= & {} \left[ \hat{A}_n(s) \exp \left( \mp \frac{\tilde{c}_{1-\alpha }^{g_1}}{\sqrt{n} \hat{A}_n(s)} \hat{\sigma }_n(s) \right) \right] _{s \in [t_1,t_2]} \nonumber \\ \textit{CB}_{\textit{HW}}^w= & {} \left[ \hat{A}_n(s) \exp \left( \mp \frac{\tilde{c}_{1-\alpha }^{g_2}}{\sqrt{n}\hat{A}_n(s)} \Big (1+\hat{\sigma }_n^2(s)\Big ) \right) \right] _{s \in [t_1,t_2]} \nonumber \\ \textit{CB}_{\textit{dir}}^w= & {} \left[ \hat{A}_n(s) \mp \frac{\tilde{c}_{1-\alpha }}{\sqrt{n}}\right] _{s \in [t_1,t_2]}. \end{aligned}$$

#### Remark 2

1. Note that the wild bootstrap quantile $\tilde{c}_{1-\alpha }$ does not require an estimate of $\phi$, thereby eliminating one possible cause of inaccuracy within the derivation of the other bands. However, the corresponding band $\textit{CB}_{dir}^w$ has the disadvantage to possibly include negative values.
2. The confidence bands are only well-defined if the left endpoint $t_1$ of the bands’ time interval is larger than the first observed event. In particular, these bands yield unstable results for small values of $\hat{A}_n(t_1)$ due to the division in the exponential function; see Lin et al. (1994) for a similar observation.
3. The present approach directly allows the construction of confidence bands for within-sample comparisons of multiple $A_1, \dots , A_k$. For instance, a confidence band for the difference $A_1 - A_2$ may be obtained via quantiles based on the conditional convergence in distribution $\hat{W}_{1n} - \hat{W}_{2n} {\mathop {\longrightarrow }\limits ^{d}} U_1 - U_2 \sim Gauss( 0, \psi _1 + \psi _2)$ in probability by simply applying the continuous mapping theorem and taking advantage of the independence of $U_1$ and $U_2$; see Whitt (1980) for the continuity of the difference functional. For that purpose, the distribution of
  4.4 $$\begin{aligned} D(t)=\sqrt{n} g(t)(\hat{A}_{1n}(t)-A_1(t)-(\hat{A}_{2n}(t)-A_2(t))), \end{aligned}$$
  with positive weight function *g* can be approximated by the conditional distribution of $\hat{D}(t)= g(t) (\hat{W}_{1n}(t)-\hat{W}_{2n}(t))$. With $g\equiv 1$, an approximate $(1-\alpha )\cdot 100\%$ confidence band for the difference $A_1 - A_2$ of two cumulative hazard functions on $[t_1,t_2]$ is
  4.5 $$\begin{aligned} \left[ \left( \hat{A}_1(s)-\hat{A}_2(s)\right) \pm \tilde{q}_{1-\alpha } / \sqrt{n}\right] _{s\in [t_1,t_2]}, \end{aligned}$$
  where
  $$\begin{aligned} \quad \tilde{q}_{1-\alpha }&= (1-\alpha ) \text { quantile of} \quad \mathfrak {L} \Big ( \sup _{s \in [t_1,t_2]} \big |\hat{W}_{1n}(s)-\hat{W}_{2n}(s) \big | \Big | \ \mathcal {C}_0 \Big ). \end{aligned}$$
  Similar arguments additionally enable common two-sample comparisons. A practical data analysis using other weight functions *g* in the context of cumulative incidence functions is given in Hieke et al. (2013).

#### Remark 3

(*Construction of confidence intervals*)

1. In particular, Theorem 3 yields a convergence result on $\mathbb {R}^m$ for a finite set of time points $\{s_1, \dots , s_m \}\subset [0,\tau ] , m \in \mathbb {N}$. Hence, using critical values $\tilde{c}_{1-\alpha }$ and $\tilde{c}_{1-\alpha }^g$ obtained from the law of the maximum $\max _{s_1, \dots , s_m}$ instead of the supremum, a variant of Corollary 1 specifies simultaneous confidence intervals $I_1 \times \dots \times I_m$ for $(A(s_1), \dots , A(s_m))$ with asymptotic coverage probability $1-\alpha$. Since the error multiplicity is taken into account, the asymptotic coverage probability of a single such interval $I_j$ for $A(s_j)$ is greater than $1-\alpha$.
2. Due to the asymptotic independence of the entries of the multivariate Nelson–Aalen estimator, a confidence region for the value of a multivariate cumulative hazard function $$(A_1(t), \dots , A_k(t))$$ at time $$t \in [0,\tau ]$$ may be found using Šidák’s correction: Letting $$J_1, \dots , J_k$$ be pointwise confidence intervals for $$A_1(t),$$$$\dots ,$$$$A_k(t)$$ with asymptotic coverage probability $$(1-\alpha )^{1/k}$$, each found using the wild bootstrap principle, the coverage probability of $$J_1 \times \dots \times J_k$$ for $$A_1(t) \times \dots \times A_k(t)$$ clearly goes to $$1 -\alpha $$ as $$n \rightarrow \infty $$.

### Hypothesis tests for equivalence, inferiority, superiority, and equality

Adapting the principle of confidence interval inclusion as discussed in Wellek (2010), Section 3.1, to time-simultaneous confidence bands, hypothesis tests for equivalence of cumulative hazard functions become readily available. To this end, let $\ell , u: [t_1,t_2] \rightarrow (0,\infty )$ be positive, continuous functions and denote by $(a_n(s),\infty )_{s \in [t_1,t_2]}$ and $[0,b_n(s))_{s \in [t_1,t_2]}$ the one-sided (half-open) analogues of any confidence band of the previous subsection with asymptotic coverage probability $1-\alpha$. Furthermore, let $A_0: [t_1,t_2] \rightarrow [0,\infty )$ be a pre-specified non-decreasing, continuous function for which equivalence to *A* shall be tested. More precisely:

$$\begin{aligned} H : \{ A(s) \le A_0(s) - \ell (s) \text { or } A(s) \ge A_0(s) + u(s) \text { for some } s \in [t_1,t_2] \} \\ \text {vs.} \quad K : \{ A_0(s) - \ell (s)< A(s) < A_0(s) + u(s) \text { for all } s \in [t_1,t_2] \}. \end{aligned}$$

#### Corollary 2

Under the assumptions of Theorem 3, a hypothesis test $\psi _n$ of asymptotic level $\alpha$ for *H* vs *K* is given by the following decision rule: Reject *H* if and only if the combined two-sided confidence band $(a_n(s),b_n(s))_{s \in [t_1,t_2]}$ is fully contained in the region spanned by $(A_0(s) - \ell (s), A_0(s) + u(s))_{s \in [t_1,t_2]}$. Further, it holds under *K* that $\mathbb {E}(\psi _n) \rightarrow 1$ as $n\rightarrow \infty$, i.e., $\psi _n$ is consistent.

Similar arguments lead to analogue one-sided tests for the inferiority or superiority of the true cumulative hazard function to a prespecified function $A_0$. Moreover, statistical tests for equality of two cumulative hazard functions can be constructed using the weak convergence results of Remark 2(c):

$$\begin{aligned} H_{=} : \{ A_1 \equiv A_2 \text { on } [t_1,t_2] \} \quad \text {vs} \quad K_{\ne } : \{ A_1(s) \ne A_2(s) \text { for some } s \in [t_1,t_2] \}. \end{aligned}$$

Corollary 3 below yields an asymptotic level $\alpha$ test for $H_{=}$. Bajorunaite and Klein (2007) and Dobler and Pauly (2014) used similar two-sided tests for comparing cumulative incidence functions in a two-sample problem.

#### Corollary 3

(A Kolmogorov–Smirnov-type test) Under the assumptions of Theorem 3 and letting *g* again be a positive weight function,

$$\begin{aligned} \varphi ^{KS}_n = \mathbf {1}\{ \sup _{s \in [t_1,t_2]} \sqrt{n} g(s) | \hat{A}_{1n}(s) - \hat{A}_{2n}(s) | > \tilde{q}_{1-\alpha } \} \end{aligned}$$

defines a consistent, asymptotic level $\alpha$ resampling test for $H_=$ vs. $K_{\ne }$. Here $\tilde{q}_{1-\alpha }$ is the $(1-\alpha )$-quantile of $\mathfrak {L} \big ( \sup _{s \in [t_1,t_2]} \big |\hat{D}(s) \big | \big | \ \mathcal {C}_0 \big )$.

Similarly, Theorem 3 enables the construction of other tests, e.g., such of Cramér-von Mises-type. Furthermore, by taking the suprema over a discrete set $\{s_1,\dots , s_m\} \subset [0,\tau ]$, the Kolmogorov–Smirnov test of Corollary 3 can also be used to test

$$\begin{aligned}&\tilde{H}_{=} : \{ A_1(s_j) = A_2(s_j) \text { for all } 1\le j\le m \} \\&\quad \text {vs.} \quad \tilde{K}_{\ne } : \{ A_1(s_j) \ne A_2(s_j) \text { for some }1\le j\le m \} . \end{aligned}$$

Note that in a similar way, two-sample extensions of Corollaries 2 and 3 can be established following Dobler and Pauly (2014).

### Tests for proportionality

A major assumption of the widely used Cox (1972) regression model is the assumption of proportional hazards over time. Several authors have developed procedures for testing the null hypothesis of proportionality, see e.g., Gill and Schumacher (1987), Lin (1991), Grambsch and Therneau (1994), Hess (1995), Scheike and Martinussen (2004), Kraus (2007), Bagdonavičius et al. (2010), or Chen et al. (2015), and the references cited therein. However, most of these approaches have mainly been investigated for the standard survival framework under (independent) right-censoring and left-truncation mechanisms, even though they may be generalized to more general settings. In the context of the proposed wild bootstrap resampling technique, this motivates the explicit formulation of a two-sample proportionality test for the general setting only assuming a multiplicative intensity structure. This covers, for instance, arbitrary Markovian multistate models.

The framework is an unpaired two-sample model given by independent counting processes $N^{(1)}, N^{(2)}$ and predictable processes $Y^{(1)}, Y^{(2)}$, assuming the conditions of Sect. 2 for each group, and with sample sizes $n_1$ and $n_2$, respectively. Let again $J^{(j)}(t) = \mathbf {1}\{ Y^{(j)}(t) > 0 \}$, $j=1,2$. Denote by $\hat{A}^{(j)}_{n_j} = \int _{(0,t]} \frac{J^{(j)}(s)}{Y^{(j)}(s)} d N^{(j)}$ the Nelson–Aalen estimator of the cumulative hazard functions $A^{(j)}$ and by $\alpha ^{(j)}$ the corresponding rates, $j=1,2$. To motivate a suitable test statistic we make use of the following equivalence between hazards proportionality and equality of both cumulative hazards:

$$\begin{aligned}&\alpha ^{(1)}(t) = c \ \alpha ^{(2)}(t) \ \text {in} \ t \in [0,\tau ] \ \text {for} \ c> 0 \\&\quad \Longleftrightarrow \ \ A^{(1)}(t) = c \ A^{(2)}(t) \ \text {in} \ t \in [0,\tau ] \ \text {for} \ c > 0 , \end{aligned}$$

which, as the null hypothesis of interest, is denoted by $H_{0,\text {prop}}$. In a natural way, similar to Gill and Schumacher (1987), this leads to statistics of the form

$$\begin{aligned} T_{n_1,n_2} = \rho \left( \ \sqrt{\frac{n_1 n_2}{n}} \frac{\hat{A}^{(2)}_{n_2}}{\hat{A}^{(1)}_{n_1}}, \ \sqrt{\frac{n_1 n_2}{n}} \frac{\hat{A}^{(2)}_{n_2}(\tau )}{\hat{A}^{(1)}_{n_1}(\tau )} \ \right) , \end{aligned}$$

$n=n_1+n_2$, where $\rho$ is an adequate distance on $\mathfrak {D}[0,\tau ]$, e.g., $\rho (f,g) = \sup w |f - g|$ (leading to Kolmogorov–Smirnov-type tests), $\rho (f,g) = \int (f-g)^2 w^2 d \lambda \!\! \lambda$ (leading to Cramér-von-Mises-type tests), where $w: [0,\tau ] \rightarrow [0,\infty )$ is a suitable weight function. Later on, we choose $w = \hat{A}^{(1)}_{n_1}$ which ensures the evaluation of $\rho$ on $\{ \hat{A}^{(1)}_{n_1} > 0 \}$. Let $\hat{W}_{n_1}^{(1)}$ and $\hat{W}_{n_2}^{(2)}$ be the obvious wild bootstrap versions of the sample-specific centered Nelson–Aalen estimators; cf. (3.2).

#### Theorem 4

Let $\rho$ be either the above Kolmogorov–Smirnov- or the Cramér-von Mises-type statistic with $w = \hat{A}^{(1)}_{n_1}$. If $n_1 / n \rightarrow p \in (0,1)$ as $\min (n_1,n_2) \rightarrow \infty$, then the test for $H_{0, prop }$

$$\begin{aligned} \varphi _{n_1,n_2}^{ prop } = \mathbf {1} \{ T_{n_1,n_2} > \tilde{q}_{1 - \alpha } \} \end{aligned}$$

has asymptotic level $\alpha$ under $H_{0,prop}$ and asymptotic power 1 on the whole complement of $H_{0,prop}$. Here $\tilde{q}_{1 - \alpha }$ is the $(1 - \alpha )$-quantile of

$$\begin{aligned}&\mathfrak {L} \left( \rho \left( \sqrt{\frac{n_1}{n}} \frac{\hat{W}_{n_2}^{(2)}}{\hat{A}_{n_1}^{(1)}} - \sqrt{\frac{n_2}{n}} \hat{W}_{n_1}^{(1)} \frac{\hat{A}_{n_2}^{(2)}}{[\hat{A}_{n_1}^{(1)}]^2} \ , \ \sqrt{\frac{n_1}{n}} \frac{ \hat{W}_{n_2}^{(2)}(\tau )}{\hat{A}_{n_1}^{(1)}(\tau )}\right. \right. \\&\left. \left. \quad - \sqrt{\frac{n_2}{n}} \hat{W}_{n_1}^{(1)}(\tau ) \frac{\hat{A}_{n_2}^{(2)}(\tau )}{[\hat{A}_{n_1}^{(1)}(\tau )]^2} \right) \Big | \mathcal {C}_0 \right) . \end{aligned}$$

## Simulation study

**Fig. 1:**
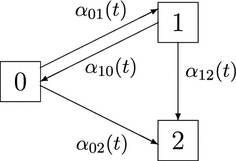
Illness-death model with recovery and transition hazards $\alpha _{01},\ \alpha _{10},\ \alpha _{02},$ and $\alpha _{12}$ at time *t*

The motivating example behind the present simulation study is the SIR-3 data of Sect. 6. The setting is a specification of Example 1(a) called *illness-death model with recovery*. As illustrated in the multistate pattern of Fig. 1, the model has state space ${\mathcal {S}}=\lbrace 0,1,2\rbrace$ and includes the transition hazards $\alpha _{01},\ \alpha _{10},\ \alpha _{02},$ and $\alpha _{12}$. The simulation of the underlying quantities is based on the methodology suggested by Allignol et al. (2011) generalized to the time-inhomogeneous Markovian multistate framework, which can be seen as a nested series of competing risks experiments. More precisely, the individual initial states are derived from the proportions of individuals at $t=0$ and the censoring times are obtained from a multinomial experiment using probability masses equal to the increments of the censoring Kaplan–Meier estimate originated from the SIR-3 data. Similarly, event times are generated according to a multinomial distribution with probabilities given by the increments of the original Nelson–Aalen estimators. These times are subsequently included into the multistate simulation algorithm described in Beyersmann et al. (2012), Section 8.2. Since censoring times are sampled independently and each simulation step is only based on the current time and the current state, the resulting data follows a Markovian structure. A more formal justification of the multistate simulation algorithm can be found in Gill and Johansen (1990) and Theorem II.6.7 in Andersen et al. (1993).

**Table 1:** Mean number of events per transition on [5, 30] provided by the simulation study of Sect. 5

Sample size	Transition			
	$1\rightarrow 0$	$0\rightarrow 1$	$0\rightarrow 2$	$1\rightarrow 2$
93	20.1	4.3	43.9	10.1
186	42.7	8.3	96.5	21.7
373	85.6	17.0	193.4	43.7
747	170.9	33.9	387.4	87.4
$747^{\mathrm{a}}$	171	34	387	87

$$^{\mathrm{a}}$$Original data

We consider three different sample sizes: The original number of 747 patients is stepwisely reduced to 373, 186, and 93 patients. For each scenario we simulate 1000 studies. As an overview, the mean number of events for each possible transition and scenario is illustrated in Table 1.

**Fig. 2:**
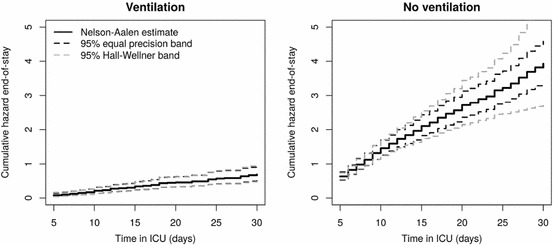
95% confidence bands based on standard normal multipliers for the cumulative hazard of end-of-stay from the data example in Sect. 6. The solid black lines are the Nelson–Aalen estimators separately for ‘no ventilation’ (state 0, right plot) and ‘ventilation’ (state 1, left plot)

The mean number of events regarding 747 patients reflects the original number of events. All numbers are restricted to the time interval [5,30], which is chosen due to a small amount of events before $t=5$ (left panel of Fig. 2). Further, less than 10% of all individuals are still under observation after day 30. In particular, asymptotic approximations tend to be poor at the left- and right-hand tails; cf. Remark 2(b) and Lin (1997).

Utilizing the R-package sde (Iacus 2014), the quantiles $c_{1-\alpha }^g$ in (4.3) of each single study are empirically estimated by simulating 1000 sample paths of a standard Brownian bridge. These quantiles are separately derived for both the Aalen- and Greenwood-type variance estimates (2.5) and (2.6). The bootstrap critical values are based on 1000 bootstrap realizations of $\hat{{{\varvec{W}}}}_n$ for each simulation step including both standard normal and centered Poisson variates with variance one. The latter is motivated by a slightly better performance compared to standard normal multipliers (Beyersmann et al. 2013; Dobler et al. 2017). Furthermore, Liu (1988) argued in a classical (linear regression) problem that wild bootstrap weights with skewness equal to one satisfy the second order correctness of the resampling approach. According to the cited simulation results, a similar result might hold true in our context, as the Poisson variates have skewness equal to one and standard normal variates are symmetric. A careful analysis of the convergence rates, however, is certainly beyond the scope of this article. In order to guarantee statistical reliability, we do not derive confidence bands for sample sizes and transitions with a mean number of observed transitions distinctly smaller than 20. The nominal level is set to $\alpha =0.05$. All simulations are performed with the R-computing environment version 3.3.2 (R Core Team 2016).

**Table 2:** Empirical coverage probabilities (%) from the simulation study of Sect. 5 separately for each transition and different simulated number of individuals

Transition	*N*	Type of confidence band									
		Brownian bridge				Wild bootstrap					
		95% log EP		95% log HW		95% log EP		95% log HW		95% direct	
		Aalen	Greenwood	Aalen	Greenwood	Poisson	Standard normal	Poisson	Standard normal	Poisson	Standard normal
$0\rightarrow 1$	373	96.4	95.9	95.5	95.3	92.5	92.5	92.5	92.5	91.4	91.9
	747	97.7	97.3	97.2	97.0	95.0	94.9	95.2	95.0	92.9	93.2
$0\rightarrow 2$	93	98.0	97.1	98.4	97.3	97.6	97.8	97.3	96.0	96.6	96.6
	186	98.3	95.5	98.9	97.4	97.2	98.2	98.0	96.1	96.2	96.2
	373	98.1	95.0	98.2	96.9	97.2	97.3	97.1	97.1	96.0	96.3
	747	98.6	96.2	98.8	97.7	97.7	97.8	97.4	97.8	96.0	96.2
$1\rightarrow 0$	93	97.0	94.9	97.0	95.2	95.1	95.1	94.8	94.8	93.7	93.7
	186	97.3	95.8	97.7	96.1	95.6	95.7	95.7	95.4	94.5	94.3
	373	97.2	96.3	97.9	97.0	95.2	95.3	95.9	96.3	95.2	95.3
	747	97.8	96.8	97.5	96.9	96.6	96.6	95.9	96.0	96.1	96.3
$1\rightarrow 2$	186	97.5	96.7	97.2	96.2	94.7	94.5	94.3	94.7	93.2	93.3
	373	98.2	97.7	98.2	97.8	95.8	95.8	95.1	95.2	94.6	94.3
	747	97.2	96.6	96.6	96.0	94.4	95.5	94.3	94.7	94.9	95.1

EP: equal-precision band; HW: Hall–Wellner band

Following Table 2, almost all bands constructed via Brownian bridges consistently tend to be rather conservative in our setting, i.e., result in too broad bands. Here, the usage of the Greenwood-type variance estimate yields more accurate coverage probabilities compared to the Aalen-type estimate. In contrast, the wild bootstrap approach mostly outperforms the Brownian bridge procedures: The log-transformed wild bootstrap bands approximately keep the nominal level even in the smaller sample sizes, except for the $0 \rightarrow 1$ transition with smallest sample size (corresponding to only 17 events in the mean; cf. Table 1). We also observe that the log-transformation in general improves coverage for the wild bootstrap procedure. The current simulation study showed no clear preference for the choice of weight. Note that all wild bootstrap bands for transition $0\rightarrow 2$ show a similar, but mostly reduced conservativeness compared to the bands provided by Brownian bridges. We have to emphasize that coverage probabilities for the cumulative hazard functions are drastically decreased to approximately 75% in all sample sizes if log-transformed pointwise confidence intervals would wrongly be interpreted time-simultaneously (results not shown).

The second set of simulations follows the test for proportional hazards derived in Theorem 4 with regard to keeping the preassigned error level under the null hypothesis. For that purpose, we assume a competing risks model with two competing events separately for two unpaired patient groups. For an illustration, see for instance, Figure 3.1 in Beyersmann et al. (2012).

We consider four different constant hazard scenarios: (I) the hazards for the type-1 event are set to $\alpha ^{(1)}_{01}(t)=\alpha ^{(2)}_{01}(t)=2$ (no effect on the type-1 hazard, in particular, a hazard ratio of $c=1$); (II) $\alpha ^{(1)}_{01}(t)=1$ and $\alpha ^{(2)}_{01}(t)=2$ (large effect); (III) $\alpha ^{(1)}_{01}(t)=\alpha ^{(2)}_{01}(t)=1$; (IV) $\alpha ^{(1)}_{01}(t)=1$ and $\alpha ^{(2)}_{01}(t)=1.5$ (moderate effect). In each scenario, we set $\alpha ^{(1)}_{02}=\alpha ^{(2)}_{02}(t)=2$, in particular, we consistently assume no group effect on the competing hazard. Further, scenario-specific administrative censoring times are chosen such that approximately 25% of the individuals are censored. The simulations designs are selected such that we include different effect sizes as well as different type-1 hazard ratio configurations with respect to the competing hazards. We consider a balanced design with $n_1=n_2=n\in \lbrace 125,250,500,1000\rbrace$. The right-hand tail of the domain of interest is set to $\tau =0.3$. Simulation of the event times and types follows the procedure explained in Chapter 3.2 of Beyersmann et al. (2012). As before, we simulate 1000 studies for each scenario and sample size configuration, whereas the critical values of the Kolmogorov–Smirnov-type and Cramér-von-Mises-type statistics from Sect. 4.3 are derived from 1000 bootstrap samples utilizing both standard normal and centered Poisson variates with variance one.

The results for the type I error rates (for $\alpha =0.05$) are displayed in Table 3. As expected from consistency, the type I error control becomes better with a larger number of patients for both test statistics in each scenario. Except for Scenario (II), all procedures keep the type I error rate quite accurately for $n\ge 500$. For smaller sample sizes, all tests tend to be conservative with a particular advantage for the Kolmogorov–Smirnov statistic.

**Table 3:** Simulated size of $\phi _{n_1,n_2}^{\text {prop}}$ for nominal size $\alpha =5\%$ under different sample sizes and constant hazard configurations

	Scenario I				Scenario II				Scenario III				Scenario IV			
	KMS		CvM		KMS		CvM		KMS		CvM		KMS		CvM	
$n_i$	SN	Poi	SN	Poi	SN	Poi	SN	CvM	SN	Poi	SN	Poi	SN	Poi	SN	Poi
125	0.029	0.024	0.033	0.030	0.029	0.026	0.027	0.023	0.045	0.041	0.028	0.030	0.046	0.042	0.030	0.025
250	0.035	0.039	0.039	0.040	0.040	0.038	0.037	0.034	0.039	0.040	0.037	0.034	0.034	0.034	0.033	0.030
500	0.057	0.054	0.059	0.060	0.034	0.038	0.040	0.041	0.056	0.053	0.047	0.045	0.044	0.044	0.043	0.044
1000	0.050	0.050	0.047	0.047	0.048	0.049	0.043	0.046	0.047	0.049	0.045	0.048	0.058	0.059	0.053	0.056

In each scenario $\tau =0.3$ and $25\%$ of individuals are censored

*KMS* Kolmogorov–Smirnov-type statistic, *CvM* Cramér-von-Mises-type statistic; *SN* standard normal multiplier; *Poi* centered poisson multiplier

## Data example

**Fig. 3:**
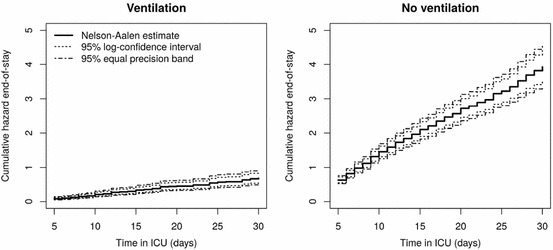
95% equal precision confidence bands based on standard normal multipliers and 95% log-transformed pointwise confidence intervals for the cumulative hazard of end-of-stay from the data example in Sect. 6. The solid black lines are the Nelson–Aalen estimators separately for ‘no ventilation’ (state 0, right plot) and ‘ventilation’ (state 1, left plot)

**Fig. 4:**
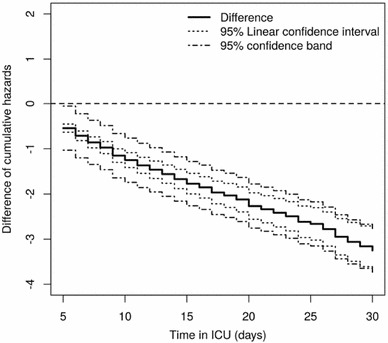
95% confidence bands from relation (4.5) based on standard normal multipliers and 95% linear pointwise confidence intervals for difference of the two cumulative hazards of end-of-stay from the data example in Sect. 6. The solid black lines is the difference ‘ventilation vs. no ventilation’ of the Nelson–Aalen estimators within the two ventilation groups

The SIR-3 (*S*pread of Nosocomial *I*nfections and *R*esistant Pathogens) cohort study at the Charité University Hospital in Berlin, Germany, prospectively collected data on the occurrence and consequences of hopital-aquired infections in intensive care (Beyersmann et al. 2006; Wolkewitz et al. 2008). A device of particular interest in critically ill patients is mechanical ventilation. The present data analysis investigates the impact of ventilation on the length of intensive care unit stay which is, e.g., of interest in cost-benefit analyses in hospital epidemiology (Beyersmann et al. 2011). The analysis considers a random subset of 747 patients of the SIR-3 data which one of us has made publicly available (Beyersmann et al. 2012). Patients may either be ventilated (state 1 as in Fig. 1) or not ventilated (state 0) upon admission. Switches in device usage are modeled as transitions between the intermediate states 0 and 1. Patients move into state 2 upon discharge from the unit. The numbers of observed transitions are reported in the last row of Table 1. We start by separately considering the two cumulative end-of-stay hazards $A_{12}$ and $A_{02}$, followed by a more formal group comparison as in Remark 2(c). Based on the approach suggested by Beyersmann et al. (2012), Section 11.3, we find it reasonable to assume the Markov property. Figure 2 displays the Nelson–Aalen estimates of $A_{12}$ and $A_{02}$ accompanied by simultaneous 95% confidence bands utilizing the 1000 wild bootstrap versions with standard normal variates and restricted to the time interval [5,30] of intensive care unit days. As before, the left-hand tail of the interval is chosen, because Nelson–Aalen estimation regarding $A_{12}$ picks up at $t=5$, cf. the left panel of Fig. 2. Graphical validation of empirical means and variances of $\hat{{\varvec{W}}}_{n}$ showed good compliance compared to the theoretical limit quantities stated in Remark 1. Bands using Poisson variates are similar (both results not shown). Figure 3 also displays the 95% pointwise confidence intervals based on a log-transformation. The performance of both equal precision and Hall–Wellner bands is comparable for transitions out of the ventilation state. However, the latter tend to be larger for the $0\rightarrow 2$ transitions for later days due to more unstable weights at the right-hand tail. Equal precision bands are graphically competitive when compared to the pointwise confidence intervals. Ventilation significantly reduces the hazard of end-of-stay, since the upper half-space is not contained in the 95% confidence band of the cumulative hazard difference, see Fig. 4.

A second investigation exemplary applies the nonparametric proportionality test suggested in Sect. 4.3 to the present study example. For that purpose, we consider sex-specific subsamples (441 male vs. 306 female patients) and test $H_{0,{ prop }}$ using both the Kolmogorov–Smirnov- and the Cramér-von-Mises-type test statistic. The Nelson–Aalen estimators separately displayed for males and females are given in Supplementary Figure S1. *p* values are computed from 1000 bootstrap samples utilizing standard normal variates and the right-hand limit of the interval of interest is set to 30 days. Tabulated results are in Supplementary Table S1 including the test statistics $T_{n_1,n_2}$ and the bootstrap quantiles $\tilde{q}_{0.95}$ of Theorem 4 as well as the corresponding bootstrap *p* values $\tilde{p}$. Non-proportionality cannot be inferred for any of the transitions at the $5\%$ level.

A concise R-script is available in the online supplementary material, which executes the bootstrap core operation given in relation (3.2) in a computationally efficient way.

## Discussion and further research

We have given a rigorous presentation of a weak convergence result for the wild bootstrap methodology for the multivariate Nelson–Aalen estimator in a general setting only assuming Aalen’s multiplicative intensity structure of the underlying counting processes. This allowed the construction of time-simultaneous confidence bands and intervals as well as asymptotically valid equivalence and equality tests for cumulative hazard functions. In the context of time-to-event analysis, our general framework is not restricted to the standard survival or competing risks setting, but also covers arbitrary Markovian multistate models with finite state space, other classes of intensity models like relative survival or excess mortality models, and even specific semi-Markov situations. Additionally, independent left-truncation and right-censoring can be incorporated. The procedure has also been used to construct a test for proportional hazards. We want to emphasize that the framework induces a random number of multipliers in relation (3.2); thus, goes beyond existing approaches for competing risks as done in Beyersmann et al. (2013) or Lin (1997). Easy and computationally convenient implementation and within- or two-sample comparisons demonstrate its attractiveness in various practical applications.

Future work will be on the approximation of the asymptotic distribution corresponding to the matrix of transition probabilities (see Aalen and Johansen 1978) and functionals thereof in general Markovian multistate models. This is of great practical interest, because no similar Brownian Bridge procedure is available to perform time-simultaneous statistical inference. In particular, previous implications rely on pointwise considerations. Note that such an approach would significantly simplify the original justifications given by Lin (1997) and generalizes his idea mainly used in the context of competing risks (Scheike and Zhang 2003; Hyun et al. 2009; Beyersmann et al. 2013). In addition, we plan to extend the utilized wild bootstrap technique to general semiparametric regression models; see Lin et al. (2000) for an application in the survival context. Current work investigates to what degree the martingale properties presented in this article may be exploited to obtain wild bootstrap consistencies for such functionals of Nelson–Aalen estimates or for estimators in semiparametric regression models. We are confident that the present approach will lead to reliable inference procedures in these contexts for which there has been only little research on such general methodology.

In contrast to the procedure of Schoenfeld et al. (2002) or the framework in Cube et al. (2017), the more general illness-death model with recovery does not rely on a constant hazards assumption and captures both the time-dependent structure of mechanical ventilation and the competing event ‘death in ICU’. This significantly improves medical interpretations. The widths of the confidence bands were competitive compared to the pointwise confidence intervals, i.e., demonstrated usefulness in practical situations.

In the present data analysis, the multistate perspective is the natural way to assess the impact of time-dependent exposures on complex survival outcomes. This is a current topic in various fields of applied research; thus, the wild bootstrap procedure in its very general formulation is practicable to analyses regarding, for instance, different stages of illicit drug use (Mayet et al. 2012), the clinical course of liver diseases (Jepsen et al. 2015), antibiotics in hospital epidemiology (Munoz-Price et al. 2016), alternative outcomes in leukemia trials (Schmoor et al. 2013; Eefting et al. 2016), or joint replacements in orthopaedic patients (Gillam et al. 2012). It has even been recently applied in a study investigating femoral fracture risk, disability, and mortality in an elderly population (Bluhmki et al. 2017).

It has to be emphasized that our simulation study suggested that the wild bootstrap approach leads to more powerful procedures (i.e., to narrower confidence bands) compared to the approximation via Brownian bridges. As expected, the applied log-transformation results in improved small sample properties compared to the untransformed wild bootstrap bands. Based on the current simulation study, however, it was difficult to clearly recommend which type of band and which type of multiplier should be used.

### Electronic supplementary material

Below is the link to the electronic supplementary material.

Supplementary material 1 (R 2 KB)

Supplementary material 2 (pdf 78 KB)

## A Proofs

### Proof (of Lemma 1)

: Due to similarity, it is enough to concentrate at the first component only; thus, we subsequently suppress the subscript ‘1’. The independence of all white noise processes immediately imply the orthogonality of all component processes. At first, we verify the martingale property; the square-integrability is obviously fulfilled since $E(G_1^2(0)) < \infty$. To this end, let $0 \le s \le t$. By measurability of the counting and the predictable process with respect to $\mathcal {C}_0$, we have

$$\begin{aligned} E(\hat{W}_n(t) \ | \ \mathcal {C}_s ) =\,&\sqrt{n} \int _{(0,t]} \frac{J(u)}{Y(u)} E( G(u) \ | \ \mathcal {C}_s) \ d N(u) \\ = \,&\sqrt{n} \int _{(0,s]} \frac{J(u)}{Y(u)} G(u) \ d N(u) \\&+ \sqrt{n} \int _{(s,t]} \frac{J(u)}{Y(u)} E(G(u)) \ d N(u) = \hat{W}_n(s) \end{aligned}$$

by the independence of $\sigma (G(u))$ and ${\mathcal {C}}_s$ for all $u > s$. Hence, the martingale property is shown.

The predictable variation process $\langle \hat{W}_{n} \rangle$ is the compensator of $\hat{W}_n^2$, i.e., we calculate

$$\begin{aligned}&E(\hat{W}_n^2(t) \ | \ \mathcal {C}_s) \\&\quad = n \left( \int _{(0,s]} \int _{(0,s]} + \int _{(s,t]} \int _{(0,s]} + \int _{(0,s]} \int _{(s,t]} + \int _{(s,t]} \int _{(s,t]} \right) E(G(u) G(v) \ | \ \mathcal {C}_s ) \\&\qquad \times \frac{J(u) J(v)}{Y(u) Y(v)}\ d N(u) \ d N(v) \\&\quad = n \left( \int _{(0,s]} \int _{(0,s]} G(u) G(v) + \int _{(s,t]} \int _{(0,s]} E(G(u)) G(v)\right. \\&\left. \qquad + \int _{(0,s]} \int _{(s,t]} G(u) E(G(v))\right. \\&\left. \qquad + \int _{(s,t]} \int _{(s,t]} E(G(u) G(v)) \right) \frac{J(u) J(v)}{Y(u) Y(v)} \ d N(u) \ d N(v) \\&\quad = n \left( \int _{(0,s]} \int _{(0,s]} G(u) G(v) \frac{J(u) J(v)}{Y(u) Y(v)} \ dN(u) d N(v)\right. \\&\left. \qquad + \int _{(s,t]} E(G^2(u)) \frac{J(u)}{Y^2(u)} \ d N(u) \right) \\&\quad = \hat{W}_n^2(s) + n \int _{(0,t]} \frac{J(u)}{Y^2(u)} \ d N(u) - n \int _{(0,s]} \frac{J(u)}{Y^2(u)} \ d N(u), \end{aligned}$$

again by the ${\mathcal {C}}_s$-measurability of *G*(*u*) for $u \le s$ and their independence for $u > s$. The second to last equality is due to the independence of *G*(*u*) and *G*(*v*) for $u \ne v$. Hence, $(\hat{W}_n^2(t) - n \int _{(0,t]} \frac{J(u)}{Y^2(u)} \ d N(u))_{t \in [0,\tau ]}$ is a martingale.

Letting $\varDelta f$ denote the jump-size process fo a càdlàg function *f*, the definition of the optional variation process yields

$$\begin{aligned} {[}\hat{W}_n ] (t)&= \sum _{0< s \le t} (\varDelta \hat{W}_n(s) )^2 = n \sum _{0 < s \le t} G^2(u) \frac{J(u)}{Y^2(u)} \varDelta N(u)\\&= n \int _{(0,t]} G^2(u) \frac{J(u)}{Y^2(u)} \ d N(u), \end{aligned}$$

where the sum is taken over all jump points of *N*. $\square$

### Proof (of Theorem 2)

[of Theorem 2]: It is enough to verify the conditions of Rebolledo’s martingale central limit theorem (in conditional probability); see e.g., Theorem II.5.1 in Andersen et al. (1993). Since the filtration ${\mathcal {C}}_0$ at time $s=0$ is not trivial, the resulting weak convergence will hold given ${\mathcal {C}}_0$ as well, in probability. From the classical theory we know that the Aalen-type variance estimator, which is in fact the predictable variation process of $\hat{W}_n$, is uniformly consistent for the variance function.

It remains to prove the Lindeberg condition (2.5.3) on page 83 in Andersen et al. (1993). But, by the same arguments as in the proof of Lemma 1, this is exactly the same as the Lindeberg condition for the Nelson–Aalen estimator itself. And this holds due to the main assumption (2.2).

Hence, Rebolledo’s martingale central limit theorem yields the desired weak convergence as well as the uniform consistency of the optional variation process. $\square$

### Proof (of Theorem 3)

[of Theorem 3]: For convergence (4.1), see Section IV.1 in Andersen et al. (1993) in combination with Slutsky’s theorem. Convergence (4.2) follows from the consistency of $\sigma ^{*2}$, Slutsky’s theorem and Theorem 2, since $\hat{W}_n$ asymptotically mimicks the distribution of $\sqrt{n} ( \hat{A}_n - A)$. The functional delta-method for $(x \mapsto \log x)$ completes the proof. $\square$

### Proof (of Corollaries 1 and 3)

[of Corollaries 1 and  3]: Due to the continuous limit distribution the conditional quantiles converge as well in probability; see e.g., Janssen and Pauls (2003), Lemma 1. The consistency of $\varphi _n^{KS}$ under $K_{\ne }$ follows from the convergence in probability of the conditional quantile towards a finite value and from the uniform consistency of the multivariate Nelson–Aalen estimator for the cumulative hazard functions. Since the factor $\sqrt{n}$ tends to infinity, the test statistic also goes to infinity in probability under $K_{\ne }$. $\square$

### Proof (of Corollary 2)

[of Corollary 2]: The proof extends the arguments of Wellek (2010), Section 3.1, from confidence intervals to confidence bands. Write $H = H_1 \cup H_2$ where

$$\begin{aligned}&H_1 : \{ A(s) \le A_0(s) - \ell (s) \text { for some } s \in [t_1,t_2] \} \\ \text {and} \quad&H_2 : \{ A(s) \ge A_0(s) + u(s) \text { for some } s \in [t_1,t_2] \}. \end{aligned}$$

Suppose *H* is true and let without loss of generality be $H_1$ true due to analogy. Then the probability of a false rejection of *H* amounts to

$$\begin{aligned}&P( A_0(s) - \ell (s)< a_n(s) \text { and } b_n(s)< A_0(s) + u(s) \text { for all } s \in [t_1,t_2] ) \\&\quad \le P( A_0(s) - \ell (s)< a_n(s) \text { for all } s \in [t_1,t_2] ) \\&\quad \le P( A(s) < a_n(s) \text { for some } s \in [t_1,t_2] ) \longrightarrow \alpha . \end{aligned}$$

Here the last inequality holds since $H_1$ is true and the convergence is due to the asymptotic coverage probability of the confidence band $(a_n(s), \infty )_{s \in [t_1,t_2]}$.

In order to prove consistency, suppose the alternative hypothesis *K* is true and choose any $\varepsilon$ such that

$$\begin{aligned} 0< \varepsilon < \inf _{s \in [t_1,t_2]} -(A_0(s) - \ell (s) - A(s)) \wedge (A_0(s) + u(s) - A(s)). \end{aligned}$$

Thus, by the (uniform) consistency of the Nelson–Aalen estimator and the wild bootstrap quantiles, the probability of a correct rejection of *H* equals

$$\begin{aligned}&P( A_0(s) - \ell (s)< a_n(s) \text { and } b_n(s)< A_0(s) + u(s) \text { for all } s \in [t_1,t_2] ) \\&\quad \ge P(A(s) - \varepsilon< a_n(s) \text { and } b_n(s) < A(s) + \varepsilon \text { for all } s \in [t_1,t_2]) \longrightarrow 1 \end{aligned}$$

as $n \rightarrow \infty$. For the convergence in the previous display, also note that $a_n \xrightarrow {\ P\ } A$ as well as $b_n \xrightarrow {\ P\ } A$ uniformly in $[t_1,t_2]$. $\square$

### Proof (of Theorem 4)

[of Theorem 4]:

Let $$t_0 > 0 $$. Denote by $$\mathfrak {D}_{>0}[t_0,\tau ] \subset \mathfrak {D}[t_0,\tau ]$$ the cone of positive càdlàg functions that are bounded away from zero. It is easy to see that the functional $$\phi : \mathfrak {D}^2_{>0}[t_0,\tau ] \rightarrow \mathfrak {D}_{>0}[t_0,\tau ], \ (f,g) \mapsto \frac{f}{g}$$ is Hadamard-differentiable tangentially to the set of pairs of continuous functions $$\mathfrak {C}^2[t_0,\tau ]$$ with continuous and linear Hadamard-derivative

$$\begin{aligned} \phi '_{(f,g)}: \mathfrak {C}^2[t_0,\tau ] \rightarrow \mathfrak {C}[t_0,\tau ], \quad (h_1,h_2) \longmapsto \frac{h_1}{g} - h_2 \frac{f}{g^2}. \end{aligned}$$

A simpler Hadamard-differentiability result holds for $\phi$’s restriction to $\tau$, i.e., $\phi |_{\tau }: (0,\infty )^2 \ni (f(\tau ), g(\tau )) \mapsto \frac{f(\tau )}{g(\tau )}$ with continuous, linear Hadamard-derivative

$$\begin{aligned} {(\phi |_\tau )}'_{(f,g)}: \mathbb {R}^2 \rightarrow \mathbb {R}, \quad (h_1(\tau ),h_2(\tau )) \longmapsto \frac{h_1(\tau )}{g(\tau )} - h_2(\tau ) \frac{f(\tau )}{g^2(\tau )}. \end{aligned}$$

Hence, we apply the functional $\delta$-method and the continuous mapping theorem to

$$\begin{aligned} \sqrt{\frac{n_1 n_2}{n}} \big (\phi \big (\hat{A}_{n_2}^{(2)}, \hat{A}_{n_1}^{(1)}\big ) - \phi \big (A^{(2)}, A^{(1)}\big )\big ) \quad \text {and} \quad \phi '_{\big (\hat{A}_{n_2}^{(2)}, \hat{A}_{n_1}^{(1)}\big )} \left( \sqrt{\frac{n_1}{n}} \hat{W}_{n_2}^{(2)}, \sqrt{\frac{n_2}{n}} \hat{W}_{n_1}^{(1)} \right) , \end{aligned}$$

respectively, verifying their equality in distribution in the limit (conditionally in probability for the latter). Proceed similarly with the restricted functional $\phi |_{\tau }$. Furthermore, the difference functional of both above functionals ensures the Hadamard-differentiability tangentially to the set of pairs of continuous functions. Our specific choices of the distance $\rho$ are continuous functionals. Hence we are able to apply the continuous mapping theorem again. To conclude the proof of the asymptotic behaviour of $\varphi _{n_1,n_2}^{ prop }$ under $H_{0,{ prop }}$, note that the particular weight function solves the problem of dividing by zero at $t_0 = 0$.

For the asymptotic power assertion, let $t_1 \in [0,\tau ]$ at which $H_{0,{ prop }}$ is violated. Then

$$\begin{aligned} \rho \Big ( \ \frac{\hat{A}^{(2)}_{n_2}}{\hat{A}^{(1)}_{n_1}} \ , \ \frac{\hat{A}^{(2)}_{n_2}(\tau )}{\hat{A}^{(1)}_{n_1}(\tau )} \ \Big ) \end{aligned}$$

converges in probability to a positive value, whence $T_{n_1,n_2} {\mathop {\rightarrow }\limits ^{p}} \infty$ follows. The conditional quantiles, however, still converge to a finite constant in probability by the above arguments. $\square$

## B Theoretical justification for the non-applicability of the ordinary multiplier resampling

In this part of the appendix, following the suggestion of a referee, we show in detail why the ordinary multiplier bootstrap fails to provide the correct limit process. For ease of presentation, we focus on the univariate Nelson–Aalen estimator in the special Markovian and uncensored multistate model: Let $(X(t))_t$ denote a Markov process with state space $\{1,2,\dots , k\}$, where $k \ge 2$, i.e., given the present state of the process, the future development does not depend on previously occupied states. Without loss of generality, we focus on the transition from state 1 to state 2 in order to prove our claim. That is, we restrict attention to the cumulative hazard function given by $A(\mathrm dt)=A_{12}(\mathrm dt) = P(X(t + \mathrm dt) = 2 \mid X(t) = 1)$; for ease of presentation, this subscript is omitted. Obviously, having shown that the competing resampling technique fails in this particular situation, the same is immediately implied in the general and multivariate case.

In this set-up, the alternative resampling approach is available because the counting process *N* for all $1 \rightarrow 2$ transitions is a sum of $n \in \mathbb {N}$ i.i.d. counting processes $N_i, i=1, \dots , n$, i.e., $N = \sum _{i=1}^n N_i$. Hence, each counting process satisfies the Doob-Meyer decomposition (2.1) such that we have the martingale representation

$$\begin{aligned} M(t) = N(t) - \int \limits _{(0,t]} Y(s) \mathrm dA(s) . \end{aligned}$$

The ordinary multiplier bootstrap works as follows: Consider the martingale representation of the Nelson–Aalen estimator,

$$\begin{aligned} W_n(t) = \sqrt{n} \int \limits _{(0,t]} \frac{J(u)}{Y(u)} \mathrm dM(u) + o_p(1) = \sqrt{n} \sum _{i=1}^n \int \limits _{(0,t]} \frac{J(u)}{Y(u)} \mathrm dM_i(u) + o_p(1), \end{aligned}$$

where $o_p(1) {\mathop {\rightarrow }\limits ^{p}} 0$ as $n \rightarrow \infty$; cf. (2.3) and omit the index *j* therein. In the resampling step, the individual counting process martingales $M_i(u)$ are replaced with $G_i \cdot N_i(u)$. Here, $G_1, \dots , G_n$ are i.i.d. random variables with zero mean and unit variance, which are independent of the data. The resulting ordinary multiplier bootstrap process shall be denoted as

$$\begin{aligned} \widetilde{W}_n(t) = \sqrt{n} \sum _{i=1}^n G_i \int \limits _{(0,t]} \frac{J(u)}{Y(u)} \mathrm dN_i(u). \end{aligned}$$

We now show that this approach asymptotically does not yield the appropriate correlation structure. The limit processes of the Nelson–Aalen estimator and its wild bootstrap version as discussed in Section 3.2 are centered Gaussian martingales and, thus, have independent increments. On the other hand, this does not hold for the limit process of $\widetilde{W}_n$: For any choice of $0 \le s \le t \le \tau$ we have

$$\begin{aligned}&cov(\widetilde{W}_n(t) , \widetilde{W}_n(s) \mid {\mathcal {C}}_0) \\&\quad = E(\widetilde{W}_n(t) \widetilde{W}_n(s) \mid {\mathcal {C}}_0) \\&\quad = n \sum _{i=1}^n \sum _{k=1}^n E( G_i G_k ) \int \limits _{(0,t]} \frac{J(u)}{Y(u)} \mathrm dN_i(u) \int \limits _{(0,s]} \frac{J(v)}{Y(v)} \mathrm dN_k(v) \\&\quad = \frac{1}{n} \sum _{i=1}^n \int \limits _{(0,t]} \frac{n J(u)}{Y(u)} \mathrm dN_i(u) \int \limits _{(0,s]} \frac{n J(v)}{Y(v)} \mathrm dN_i(v) \\&\quad = \frac{1}{n} \sum _{i=1}^n \int \limits _{(0,t]} \frac{1}{y(u)} \mathrm dN_i(u) \int \limits _{(0,s]} \frac{1}{y(v)} \mathrm dN_i(v) + o_p(1) \\&\quad {\mathop {\rightarrow }\limits ^{p}} \widetilde{\psi }(s,t) := E \left( \int \limits _{(0,t]} \frac{1}{y(u)} \mathrm dN_1(u) \int \limits _{(0,s]} \frac{1}{y(v)} \mathrm dN_1(v) \right) , \end{aligned}$$

where the last convergence in probability holds due to the law of large numbers as $n \rightarrow \infty$. Let *X* denote the Markov process underlying the counting process $N_1$. A twofold application of Fubini’s theorem yields that the above expectation equals

$$\begin{aligned} \int \limits _{(0,t]} \int \limits _{(0,s]} \frac{1}{y(u)} \frac{1}{y(v)} E ( \mathrm dN_1(u) \mathrm dN_1(v)) . \end{aligned}$$

As we assumed that there is no censoring, we have

$$\begin{aligned} E \Big ( \mathrm dN_1(u) \mathrm dN_1(v) \Big )&= P(X(u) = 1, X(u + du) = 2, X(v) = 1, X(v + dv) = 2). \end{aligned}$$

Now, we distinguish between two different cases: First, we treat the diagonal which contributes the following integral to the asymptotic covariance:

$$\begin{aligned} \int \limits _{(0, \min (s,t)]} \frac{ E(\mathrm dN_1(u) ) }{y^2(u)} = \int \limits _{(0, \min (s,t)]} \frac{ E( Y_1(u) \mathrm dA(u) ) }{y^2(u)} = \int \limits _{(0, \min (s,t)]} \frac{\mathrm dA(u) }{y(u)}, \end{aligned}$$

where $Y_1$ is the at risk indicator of the process *X* for a transition out of state 1. This integral corresponds to the asymptotic covariance function of the Nelson–Aalen estimator, cf. Theorem 1. Unfortunately, the overall asymptotic covariance of the alternative multiplier approach is increased due to the off-diagonal integral parts:

$$\begin{aligned}&\int \limits _{(0,t]} \int \limits _{(0,s]} \frac{\mathbf {1}\{u \ne v\}}{y(u) y(v)} E \Big ( \mathrm dN_1(u) \mathrm dN_1(v) \Big ) \\&\quad = \int \limits _{(0,s]} \int \limits _{(0,s]} \frac{\mathbf {1}\{u \ne v\}}{y(u) y(v)} E \Big ( \mathrm dN_1(u) \mathrm dN_1(v) \Big )\\&\qquad + \int \limits _{(s,t]} \int \limits _{(0,s]} \frac{\mathbf {1}\{u \ne v\}}{y(u) y(v)} E \Big ( \mathrm dN_1(u) \mathrm dN_1(v) \Big )\\&\quad = 2 \int \limits _{(0,s]} \int \limits _{(u,s]} \frac{1}{y(u) y(v)} E \Big ( \mathrm dN_1(u) \mathrm dN_1(v) \Big )\\&\qquad + \int \limits _{(s,t]} \int \limits _{(0,s]} \frac{1}{y(u) y(v)} E \Big ( \mathrm dN_1(u) \mathrm dN_1(v) \Big ) \end{aligned}$$

In the case of $u < v$ and due to the assumed Markov property, the above expected values reduce to

$$\begin{aligned}&E ( \mathrm dN_1(u) \mathrm dN_1(v) ) = P(X(u) = 1, X(u + du) = 2, X(v) = 1, X(v + dv) = 2) \\&\quad = P(X(v + dv) = 2 \mid X(v) = 1) \ P(X(v) = 1 \mid X(u + du) = 2) \\&\qquad \times P(X(u + du) = 2 \mid X(u) = 1 ) \ P(X(u) = 1) \\&\quad = P(X(u) = 1) \ P_{21}(u,v) \ \mathrm dA(u) \ \mathrm dA(v), \end{aligned}$$

where $P_{21}(u,v) = P(X(v) = 1 \mid X(u) = 2)$. All in all, using $y(u) = P(X(u) = 1)$, we obtain the following asymptotic covariance of the ordinary multiplier bootstrap version of the Nelson–Aalen estimator:

$$\begin{aligned} \widetilde{\psi }(s,t)= \int \limits _{(0,\min (s,t)]} \frac{\mathrm dA(u)}{y(u)} + \int \limits _{(0,s]} \int \limits _{(0,t]} \frac{P_{21}( \min (u,v), \max (u,v) )}{y( \max (u,v) ) } \mathrm dA(u) \mathrm dA(v). \end{aligned}$$

We may conclude two things from this covariance representation: First, we see that $\widetilde{\psi }(s,t) \ne \psi (s,t)$. That is, the asymptotic covariance function of the Nelson–Aalen estimator as given in Theorem 1 is altered by the ordinary multiplier bootstrap. As $\widetilde{\psi }(s,t)$ is in general not a function of $\min (s,t)$, the underlying limit Gaussian process does not even have independent increments. Hence, the alternative multiplier approach fails in reproducing an adequately distributed stochastic process.

Second, we see that the correct limit covariance structure is ensured only if the second integral in the above representation of $\widetilde{\psi }$ vanishes. This is the case if $P_{21} \equiv 0$ which holds, for example, if there is at most one $1 \rightarrow 2$ transition possible as in the competing risks special case. This is no surprise, though: In the competing risks set-up, both resampling options, our proposed wild bootstrap and the ordinary multiplier bootstrap, reduce to the same procedure; see Beyersmann et al. (2013) for the applicability of the wild bootstrap to Aalen-Johansen estimators in competing risks models.
