# Supplementary material for: The wild bootstrap for multivariate Nelson–Aalen estimators
Source: Lifetime Data Anal. 2018 Mar 6;25(1):97–127. doi: 10.1007/s10985-018-9423-x (PMC6323102; doi:10.1007/s10985-018-9423-x)
Supplement: Supplementary file 2 — Supplementary material 2 (pdf 78 KB) [file 10985_2018_9423_MOESM2_ESM.pdf]

Web-based Supplementary Material for 'The Wild Bootstrap for Multivariate Nelson-Aalen Estimators'

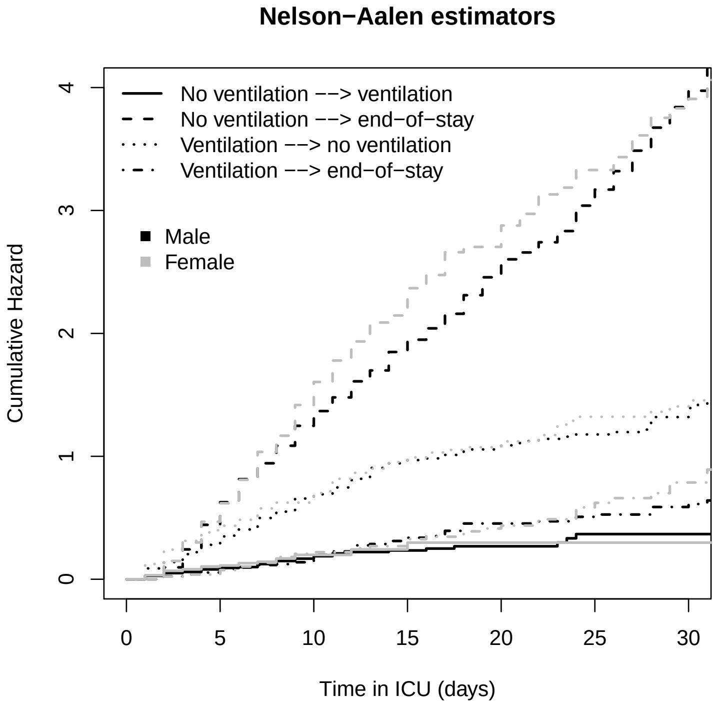

Figure S1: Nelson-Aalen estimators for each of the four possible transitions of the data example of Section 6. We separately plotted the estimators for men (black) and women (gray) restricted to the time interval of interest $[0, 30]$.

Table S1: Proportionality test statistics $T_{n_1;n_2}$ as well as the corresponding bootstrap 95% quantiles $\tilde{q}_{0.95}$ and the corresponding p-values $\tilde{p}$ resulting from applications to the data example of Section 6 (male vs. female). Quantiles are derived from 1000 bootstrap samples utilizing standard normal variates and restricted to the domain of interest $[0, 30]$ and p values are not adjusted for multiplicity.

	KMS			CvM		
	T_{n_1,n_2}	$\tilde{q}_{0.95}$	$\tilde{p}$	T_{n_1,n_2}	$\tilde{q}_{0.95}$	$\tilde{p}$
No ventilation $\rightarrow$ ventilation	1.45	1.68	0.12	16.58	21.15	0.10
No ventilation $\rightarrow$ end-of-stay	7.21	8.28	0.12	442.82	585.88	0.09
Ventilation $\rightarrow$ no ventilation	1.43	3.83	0.87	16.21	150.32	0.80
Ventilation $\rightarrow$ end-of-stay	2.60	3.19	0.15	44.42	94.20	0.20
